# Stable and Oriented Liquid Crystal Droplets Stabilized by Imidazolium Ionic Liquids

**DOI:** 10.3390/molecules26196044

**Published:** 2021-10-05

**Authors:** Efthymia Ramou, Guilherme Rebordão, Susana I. C. J. Palma, Ana C. A. Roque

**Affiliations:** 1 Associate Laboratory i4HB—Institute for Health and Bioeconomy, School of Science and Technology, NOVA University Lisbon, 2829-516 Caparica, Portugal; e.ramou@fct.unl.pt (E.R.); g.rebordao@campus.fct.unl.pt (G.R.); s.palma@fct.unl.pt (S.I.C.J.P.); 2 UCIBIO—Applied Molecular Biosciences Unit, Department of Chemistry, School of Science and Technology, NOVA University Lisbon, 2829-516 Caparica, Portugal

**Keywords:** microfluidics, liquid crystal droplets, imidazolium ionic liquids

## Abstract

Liquid crystals represent a fascinating intermediate state of matter, with dynamic yet organized molecular features and untapped opportunities in sensing. Several works report the use of liquid crystal droplets formed by microfluidics and stabilized by surfactants such as sodium dodecyl sulfate (SDS). In this work, we explore, for the first time, the potential of surface-active ionic liquids of the imidazolium family as surfactants to generate in high yield, stable and oriented liquid crystal droplets. Our results show that [C_12_MIM][Cl], in particular, yields stable, uniform and monodisperse droplets (diameter 74 ± 6 µm; PDI = 8%) with the liquid crystal in a radial configuration, even when compared with the standard SDS surfactant. These findings reveal an additional application for ionic liquids in the field of soft matter.

## 1. Introduction

Dynamic self-assembled architectures, as liquid crystal droplets, are extremely interesting systems to provide low energy transducers in sensing. Liquid crystals (LCs) are a class of soft materials that blend long-range order and fluidity [1,2,3]. They possess many attractive properties, namely their sensitivity to external stimuli—e.g., electrical and magnetic fields, light, temperature, chemical analytes—that result in a change in their director profile [4,5]. LC-based sensors for chemical analytes have been used in two main formats: (i) LCs deposited onto chemically-modified solid interfaces and (ii) LCs in aqueous interfaces, namely LC droplets or thin films [4,6].

Droplet systems are particularly interesting due to the facility to control their geometry in a reproducible manner using, for example, microfluidic systems. LC ordering within droplets is a result of their anisotropic elasticity and surface anchoring properties. Specific anchoring conditions can be promoted by the addition of surfactants, lipids, proteins, or polymers at the LC/water interface [4,7]. So far, LC droplets have been mainly reported using surfactants, namely sodium dodecyl sulfate (SDS), that minimize the interfacial tension between the two phases—thus facilitating droplet generation and preventing coalescence—while simultaneously promoting LC director alignment. A variety of director profiles exist, either stable or metastable, while some of them are considered intermediates to radial (homeotropic anchoring) and bipolar (planar anchoring) configurations [8,9]. The orientation of the LC is governed by tail-LC interactions between the surfactant and the LC. On the other hand, the contribution of the hydrophilic head with regards to the LC ordering is rather negligible [10,11]. It is well known that the anchoring of LCs in contact with aqueous surfaces containing surfactants is strongly influenced by the structure of the hydrophobic aliphatic chain, its length and conformation.

In this work, we explore surface active ionic liquids (ILs) as alternative agents to promote the formation of monodisperse and stable LC-droplets with defined director orientation. ILs consist of a combination of organic cations and organic (or inorganic) anions, allowing for countless possibilities of potential structural combinations. Surface active ionic liquids typically bear an amphiphilic character with a hydrophilic headgroup and a hydrophobic tail [12,13]. Here, different ILs of the 1-alkyl-3-methylimidazolium chloride family ([C_n_MIM][Cl]), and the typical SDS surfactant for comparison purposes, were studied to produce LC (4-Cyano-4’-pentylbiphenyl; 5CB) droplets via microfluidics. Different parameters were tested, such as the alkyl chain length of the ionic liquid cation, respective concentration, and the ratio (R) of the continuous phase flow rate to the disperse phase flow rate, in order to assess the effect on droplet size and stability, as well as LC anchoring and resulting director orientation.

## 2. Results

### 2.1. Impact of Ionic Liquid Concentration and Alkyl Chain Size on the Production of 5CB Droplets

**Figure 1:**
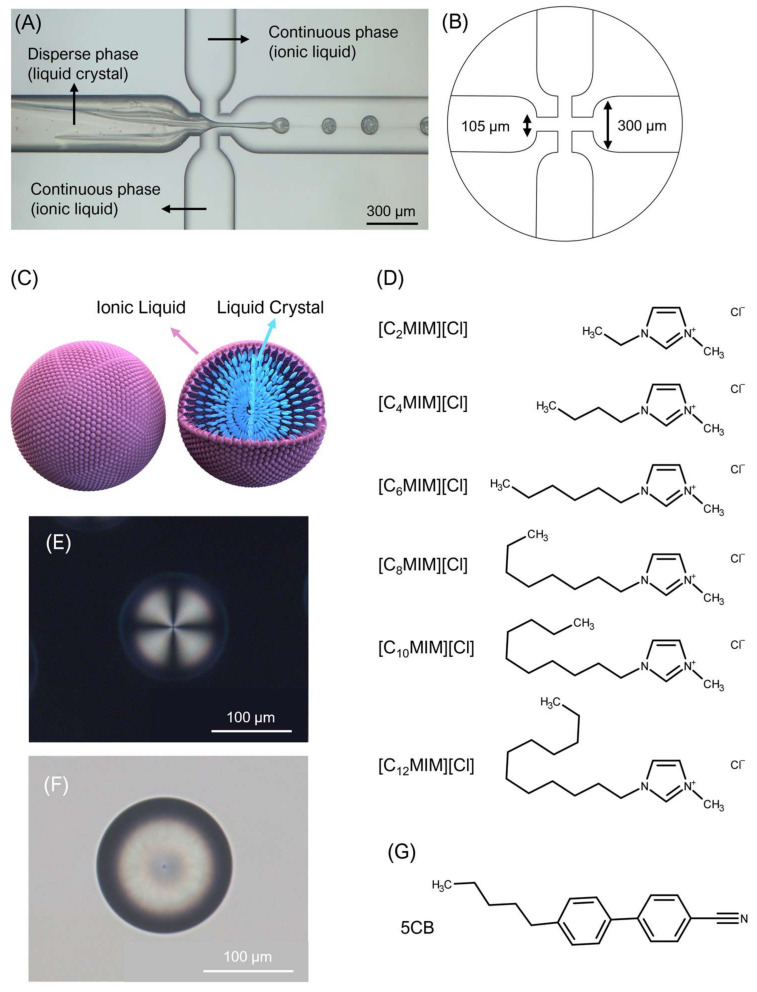
Production of liquid crystal droplets by microfluidics: (**A**) Image of droplet formation in a flow-focusing microfluidics circuit, using an ionic liquid as continuous phase and a liquid crystal as disperse phase; (**B**) Dimensions of the flow-focusing junction; (**C**) Illustration of an ionic liquid/liquid crystal radial droplet; (**D**) Molecular structure of the different ionic liquids of the imidazolium chloride family used in this work; (**E**) Polarizing optical microscopy image of a radial liquid crystal droplet taken with crossed polarizers; (**F**) Corresponding bright field image; (**G**) Molecular structure of 5CB.

Oil-in-water microdroplets were prepared via microfluidics, with 5CB—a room temperature nematic liquid crystal—being the oil phase and imidazolium-based ILs being the aqueous phase. It is well known that in [C_n_MIM][X] ILs, the surface tension (σ) decreases as the number of carbon atoms (n) in the alkyl chain of the cation increase [14]. We were interested specifically in producing radial droplets, since previous studies have revealed that radial droplets are superior to bipolar in potential sensing applications, as they respond faster to the presence of analytes [15,16,17] (See Figure 1 for details on the setup, material molecular structures and a representation of the desired droplet configuration).

**ILs with short alkyl chains.** The tested ILs [C_2_MIM][Cl] (two carbons), [C_4_MIM][Cl] (four carbons) and [C_6_MIM][Cl] (six carbons) were found to exhibit rather poor surfactant properties. In fact, it was not possible to find any data related to their estimated critical micellar concentration (CMC). For the three selected concentrations (0.02 M, 0.2 M and 0.4 M) and the seven flow rate ratios investigated for each concentration, no droplets were formed for any of the studied ILs. Instead, a separation of the two phases (liquid crystal and ionic liquid) was observed, as seen in Figure S1. It should be noted that it was quite difficult to keep the R constant when testing these ionic liquids, a finding that also reflects their weak surfactant properties [14]. The IL [C_4_MIM][DCA] (four carbons) was also included in our study as it has been previously shown that this IL produces stable 5CB droplets by emulsion and in the presence of gelatin [15,16]. However, at the concentrations tested, it was not possible to form LC droplets by microfluidics.

**ILs with long alkyl chains.** The ILs [C_8_MIM][Cl], [C_10_MIM][Cl] and [C_12_MIM][Cl] are known to possess higher surfactant potential, as denoted by their reported CMC values, 0.23 M, 0.058 M and 0.014 M, respectively [12,18]. The overall results on our investigations can be found tabulated in Table 1.

Testing [C_8_MIM][Cl] at a 0.02 M concentration resulted in phase separation, similarly to the previously discussed short chain ILs. At this concentration (lower than the CMC) [C_8_MIM][Cl] behaves like a salt and interacts preferentially with water molecules [19], which could explain the absence of droplets formation. However, by raising the concentration to 0.2 M and 0.4 M, it was possible to investigate different flow ratios. At 0.2 M and as the flow ratio increases, the produced droplet diameters decrease in an almost linear fashion, with values ranging from 190 μm to 90 μm (see ESI Figure S2). A similar trend was observed for the 0.4 M concentration; however, the droplet diameter values reach a plateau after a flow ratio R = 50 and at ~100 μm. 

It should be noted that for the flow ratio R = 5 in both concentrations, droplet formation did not occur. As mentioned in the Materials and Methods Section, the two outer inlets of the microfluidics chip were used for the IL (continuous phase) and the middle inlet was used for the LC (disperse phase). In flow-focusing geometry, by properly adjusting the flow rates of the continuous and disperse phases, the three streams enter the junction as three parallel laminar liquid streams flowing side by side without turbulence. When the flow rate of the continuous phase is increased at a given disperse phase flow rate, the disperse phase starts becoming stretched between the two opposite streams of the continuous phase [20]. This is a result of the continuous phase applying pressure and viscous stresses which force the disperse phase to become narrower [21] and form into a thread or a jet (jetting regime). Further increase in the continuous phase flow rate eventually leads to a critical point where the disperse phase thread becomes unstable and breaks, resulting in a periodic release of droplets into the outlet channel [20] (dripping regime). Here, we maintained the disperse phase flow rate at 1 µL/min and gradually increased the flow rate of the continuous phase, in order to increase R. For [C_8_MIM][Cl] at 0.2 M and at 0.4 M with R = 5, the system was likely still in the jetting regime—the disperse phase (5CB) thread was not sufficiently unstable to break—and therefore, no droplets formed. At these concentrations for [C_8_MIM][Cl] (0.2 and 0.4 M) and a 1 µL/min flow rate for 5CB, a higher ionic liquid flow rate (roughly 20 µL/min) was needed to cause the release of 5CB droplets.

Regarding the LC director profile within the droplets, neither of the two concentrations (0.2 and 0.4 M) resulted in a dominant LC configuration (ESI Figure S3). The produced droplets exhibited a mixture of radial (exhibiting the typical Maltese cross pattern in POM), escaped radial (exhibiting a distorted Maltese cross pattern with an off-center defect point, resulting from a tilted surface alignment) and axial (without a line disclination [22], resulting also from a tilted surface alignment) configurations. Furthermore, the droplets were very polydisperse, with the PDI ranging from 23–42% across the different flow ratios for the 0.2 M concentration and from 25–66% for the 0.4 M concentration. This finding is reflected in Figure S3 in the ESI, where a rather wide variability of droplet size is observed, for all the depicted conditions. Regarding the droplet yield, neither of the two tested concentrations show any trend with the increase of R, ranging from 2–28 droplets/mm^2^ for 0.2 M and from 0 to 21 droplets/mm^2^ for 0.4 M (across the different Rs). 

For both [C_10_MIM][Cl] concentrations of 0.02 M and 0.2 M, respectively, it was not possible to produce any LC droplets. At these concentrations, the disperse phase flow rate was very difficult to manipulate while keeping the 5CB flow rate at 1 µL/min. More specifically, an increase in the [C_10_MIM][Cl] flow rate would result in a decreased 5CB flow rate; hence, a stable R was not possible to acquire. We tried to adjust the R at a higher 5CB flow rate, but the same instability was observed. Nonetheless, in the few seconds right before the instability occurred, where we could reach the desired R values, there was no droplet formation. POM analysis of a sample of the outlet material, showed birefringent areas (5CB) segregated from non-birefringent areas ([C_10_MIM][Cl]). This suggests that under these conditions, [C_10_MIM][Cl] and 5CB co-flow as two immiscible phases through the outlet microchannel and higher Rs would be required to reach and overcome the jetting regime, leading to a consequent breakup of the 5CB disperse phase stream into droplets. 

When the IL concentration is increased to 0.4 M, the interfacial tension between the two phases decreases, which facilitates droplet formation [23] within the tested flow rate ratios. The size distribution varies between 85–131 μm but without showing any trend with increasing R (ESI Figure S4). The formed droplets were polydisperse and did not show a prevalent LC configuration at any R (see ESI Figure S5), but rather a mixture of different director organizations. The PDI decreases with increasing R from 55% at R = 5 to 15% at R = 95. Finally, the droplet yield across the different ratios was low, at approximately 3 droplets per mm^2^ on average for the different Rs. These findings could be attributed to the high tendency of [C_10_MIM][Cl] to self-assemble in aqueous solutions, forming predominantly spherical micelles between the CMC and 25 wt% (0.6 M) [22].

**Figure 2:**
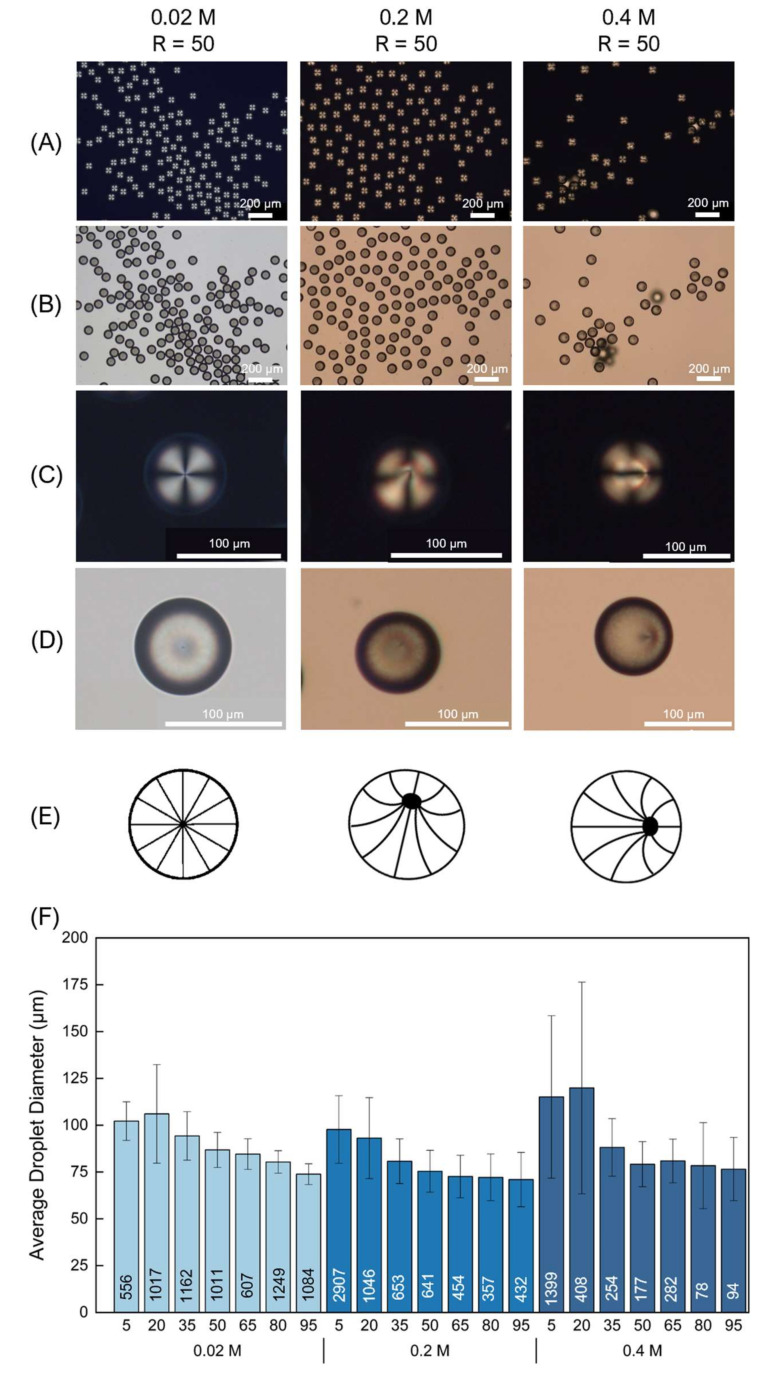
Liquid crystal droplet morphology and average size diameter results obtained when using [C_12_MIM][Cl] at concentrations 0.02 M, 0.2 M and 0.4 M, respectively. Here, results for a flow ratio R = 50 are depicted: (**A**) Polarized optical microscopy images taken with crossed polarizers; (**B**) Corresponding bright field images; (**C**) Corresponding polarized optical microscopy micrographs of single droplet close-ups; (**D**) Corresponding bright field images; (**E**) Schematic representations of the observed LC director profiles; (**F**) Droplet diameter comparison at concentrations 0.02 M, 0.2 M and 0.4 M, respectively. On the x axis the different tested flow ratios are represented. The number of analyzed droplets are shown inside the respective bars.

The last imidazolium-based IL tested was [C_12_MIM][Cl], with an alkyl chain of 12 carbon atoms. This is a solid IL at room temperature. Droplet production was achieved across all selected concentrations and flow ratios. In all the tested concentrations, the droplet diameter decreases with increasing R (see Figure 2) with values ranging from 120 μm to ~70 μm. The LC configuration within the droplets varies throughout the three tested concentrations (see Figure 2). At 0.02 M, the majority of the droplets exhibited a radial director profile across all the selected Rs. Increasing the concentration at 0.2 M, no prevalent configuration was detected, instead, a mixture of radial and escaped radial droplets was observed across all tested Rs. Finally, at 0.4 M, the majority of the droplets presented an escaped radial profile. From the POM images in Figure 2, it is evident that as the concentration of [C_12_MIM][Cl] increases, the droplet polydispersity also increases, while the LC gradually loses the radial organization. 

This abovementioned could be related to the Ostwald ripening phenomenon, a process where larger droplets grow at the expense of smaller droplets due to the transport of dispersed phase molecules from smaller droplets to larger ones [24,25]. As in any emulsion formation, the surfactant molecules are distributed between the aqueous phase and the surface of the droplets, which are not saturated with surfactant at low concentrations. As the surfactant concentration increases, the droplet surface becomes saturated and excess surfactant molecules will form micelles in the continuous phase, increasing the amount of oil phase that can be solubilized in it [24]. This increase of solubility of the dispersed phase in the continuous phase is the driving force for the Ostwald ripening process, which increasingly enhances the polydispersity of the droplets [24,25,26]. It could be hypothesized that during this process, the transfer of oil phase from the droplet to a new micelle [25] might disturb the equilibrium at the droplet surface and disrupt the radial configuration of the droplet, resulting in a escaped radial orientation.

Regarding the variability of the droplet sizes, for the 0.02 M concentration, the PDI values decrease with increasing R, from 24–8%. This finding indicates that as the flow rate of the continuous phase increases (while the flow rate of the disperse phase remains steady), the droplets become more monodisperse. Typically, when the continuous phase flow rate is increased while keeping the disperse phase flow rate constant, droplets start to form beyond a critical flow rate ratio, passing by a sequence of formation regimes. Probably due to an unstable interplay between the interfacial tension and viscous stress for lower Rs, droplet generation frequency is also unstable, leading to polydisperse droplets [20,23]. As the flow rate of the continuous phase increases (hence, R increases), the viscous thread of 5CB is subjected to a faster and periodic breakup, producing more monodisperse droplets [20]. The droplet yields present values from 40–101 droplets/mm^2^ for low values of R (R = 5) and decreases dramatically at higher R values. The decrease in droplet yield with the increase of R was anticipated since the flow rate of the disperse phase (5CB) remained steady as the flow rate of the continuous phase increased. At higher Rs (e.g., R = 95), the disperse phase is progressing slowly in the central channel, when compared to the fast progression of the continuous phase in the lateral channels. Therefore, the time interval between each droplet formation becomes larger, i.e., droplet generation frequency decreases, leading to a lower yield [20]. 

Upon increasing the [C_12_MIM][Cl] concentration at 0.2 M, the size variability of the produced droplets, across all tested Rs, exhibited a rather narrow distribution with values between 15–23%. The 0.2 M concentration facilitated a high droplet yield at low flow ratios and gradually decreased as the R reached the value of 95 (70 droplets/mm^2^ for R = 5 and 10 droplets/mm^2^ for R = 95). A similar yield trend was observed at 0.4 M, high droplet yield at lower ratios (e.g., 101 droplets/mm^2^ for R = 5), which decrease as the R reaches higher values (e.g., 7 droplets/mm^2^ for R = 95). The observed droplet yield trends are in accordance with the expected lower droplet production rates at high Rs for the three tested [C_12_MIM][Cl] concentrations. Regarding PDI, the decreasing trend observed for the 0.02 M concentration was not detected for the 0.2 M and 0.4 M concentrations. In fact, the 0.4 M concentration resulted in the most polydisperse samples. Probably, for these concentrations, larger Rs are required in order to generate more monodisperse droplets.

For comparison purposes, we also tested SDS, a well-known anionic surfactant with a reported CMC of 0.0082 M, and commonly used to promote homeotropic alignment in LC droplets [7,27,28]. Similar to [C_12_MIM][Cl], it possesses a 12-carbon alkyl chain. The surfactant was tested on the microfluidics setup for the 0.02 M and 0.2 M concentrations across all the selected Rs. For the former concentration, the produced droplet diameters decrease with increasing R with diameter values between 107–48 μm (Figure S6 in the ESI). For the latter, a decrease is also observed initially with increasing R; however, the diameter values almost stabilize at ~80μm after a flow rate ratio of R = 50. This was fairly unexpected since, as mentioned earlier, increasing the flow rate ratio R typically results in a droplet diameter decrease. Certainly, different conditions tested (i.e., different IL concentration or different Rs) could lead to alternative outcomes. However, testing all these various parameters with SDS was not within the scope of our work; hence, the droplet diameter stabilization is just reported as an experimental finding. 

With respect to the LC director profiles, in the case of the 0.02 M concentration (see Figure S7 for microscope images), the droplets exhibited a predominantly radial configuration with increasing R until the value R = 65. Beyond this R, the resulting droplets started exhibiting a prevalent escaped-radial organization. However, this was not the case for the second tested concentration of 0.2 M, the produced LC droplets presented a mix of radial and escaped-radial configurations across all tested Rs. This was very similar to the [C_12_MIM][Cl] case, since increasing the surfactant concentration had such an effect on the LC organization within the droplets. We hypothesize that it could be another manifestation of the Ostwald ripening phenomenon.

In terms of the droplet size variability, for the 0.02 M concentration, the PDI does not show a specific trend with increasing R and acquires values ranging from 7–24%. The droplet yields exhibit a decreasing trend with increasing R, presenting values from 60–11 droplets/mm^2^. At a 0.2 M SDS concentration, the PDI values show a downwards trend with increasing R between 33–8%, whereas the corresponding yields presented an almost linear dependence on increasing R (28–7 droplets/mm^2^). Although SDS has been reported to enable the production of microfluidics-based monodisperse droplets with low PDI (around 1%) in flow-focusing geometries [20], in our system and with the tested conditions, such monodispersity was not obtained. Several parameters influence droplet production, namely the viscosity and surface tension characteristics of the disperse and continuous phase, corresponding flow rates, the dimensions of the microchannels and the geometry of the channel intersection. In our system, [C_12_MIM][Cl] enabled better PDIs than SDS. Although both molecules possess 12 carbon atoms in their hydrophobic chains, they exhibit distinct surfactant properties ([C_12_MIM][Cl]: CMC = 0.014 M; SDS: CMC = 0.0082 M) and different chemical functionalities in their hydrophilic headgroup (cationic and aromatic headgroup for [C_12_MIM][Cl]; anionic, small aliphatic headgroup for SDS). Apparently, the chemistry of [C_12_MIM][Cl] facilitates the stabilization of the 5CB disperse phase, when compared to SDS under the specific conditions used here.

**Figure 3:**
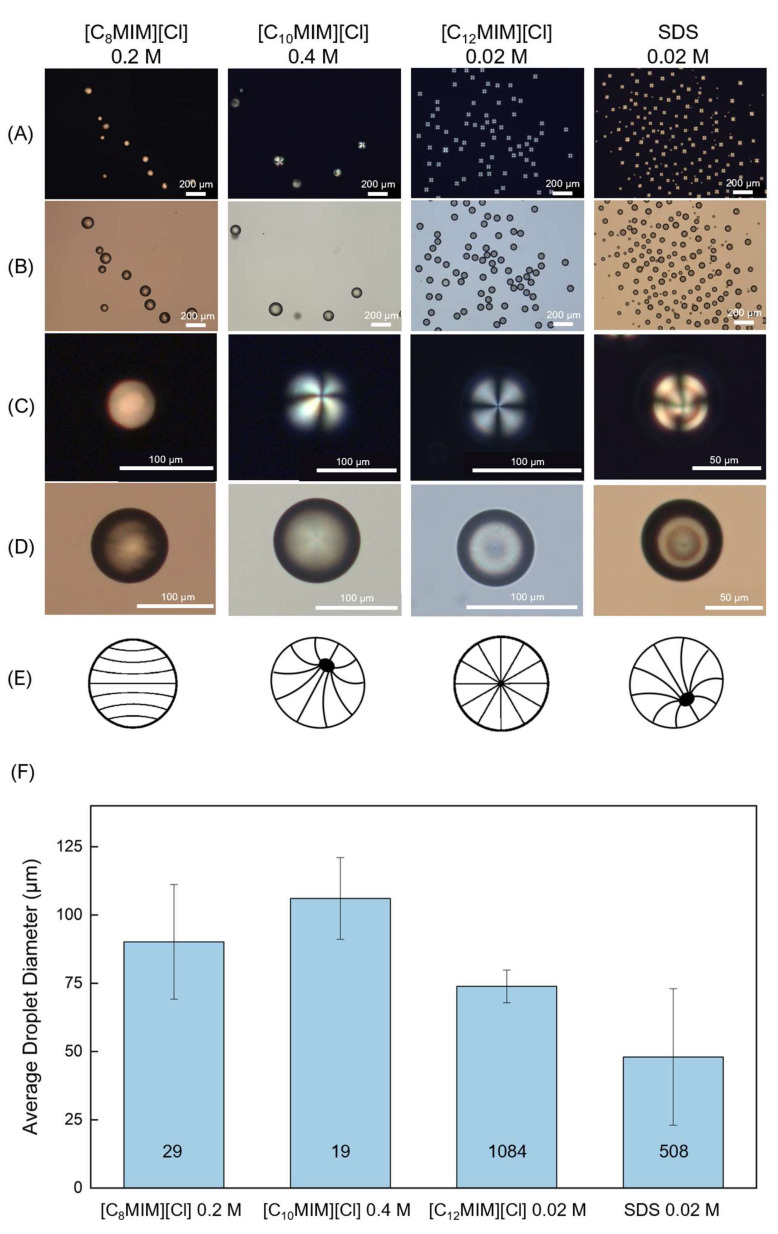
Morphology and diameter value of the liquid crystal droplets obtained when using the minimal concentrations required for droplet production, for each tested IL and SDS, for a flow rate ration R = 95: (**A**) Polarized optical microscopy photos taken with crossed polarizers; (**B**) Corresponding bright field micrographs; (**C**) Corresponding single droplet close-up (crossed polarizers); (**D**) Corresponding single droplet close-up (bright field); (**E**) Schematic representation of the various LC director profiles observed in the produced droplets (axial without a line disclination, escaped radial, radial and escaped radial, respectively); (**F**) Size comparison between the droplets produced with R = 95 for each IL. The number of analyzed droplets are shown inside the respective bars.

As a final comment on the presented findings, it can be concluded that utilizing [C_12_MIM][Cl] at a concentration of 0.02 M with a flow ratio of R = 95 provided the best results regarding droplet yield, droplet size dispersity and LC director profile. For comparison purposes, in Figure 3, POM photos of droplet profiles are depicted at a flow ratio of R = 95 for all the tested surfactants when using the minimum concentration necessary for droplet production. Additionally, a corresponding droplet size comparison graph is presented.

### 2.2. Storage Stability of 5CB Droplets

For most applications, the stability of droplets and emulsions upon storage is an important factor [29]. Typically, LC emulsions undergo coalescence, Ostwald ripening (mentioned earlier) and sedimentation onto the supporting substrate, sometimes even minutes after production. Sedimentation of LC droplets can result in director field changes but also droplet shape deformations [30,31]. Obviously, these factors affect short-term and long-term stability, for drug delivery or applications on an industrial level [32]. In this work, stability experiments were performed with [C_12_MIM][Cl] and SDS, the two surfactants that facilitated the production of radial LC droplets and at a concentration of 0.02 M. Flow rate ratio values of R = 20 and R = 95 were selected for both surfactants. This experiment served to observe the evolution of droplet stability over 60 min, by tracking the average droplet diameter and its standard deviation. More details on the procedure followed can be found in the Materials and Methods section.

**Figure 4:**
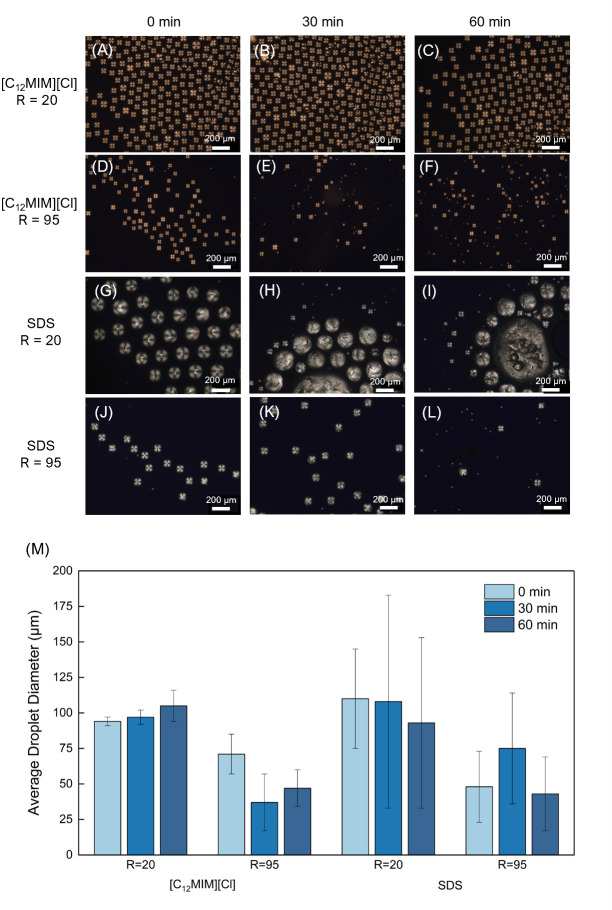
Stability experiment: (**A**–**L**) Analysis of the variation of droplet size and morphology between the moment of formation, 30 min after formation and 60 min after formation. Results for [C_12_MIM][Cl] 0.02 M and SDS 0.02 M. Only the ratios of R = 20 and R = 95 are presented; (**M**) Average droplet diameter and deviation for the three time periods.

In the case of LC droplets formed with [C_12_MIM][Cl] at concentration 0.02 M with R = 20, the PDI changed from 3% at production time, to 5% at the 30 min mark. After the one-hour mark passed, the polydispersity index increased to 11%. Interestingly, the LC organization within the droplets did not suffer any severe changes, as can be seen in Figure 4, since it remained predominantly radial. These results are quite promising, indicating that the droplets can be stored for over 30 min after being produced, before being extracted for other purposes. In the case of a flow ratio R = 95, a number of the stored droplets experienced Ostwald ripening resulting to smaller droplets being formed, while others coalesced with neighboring droplets, giving rise to larger droplet formation. This ultimately resulted in a PDI value of 53% after 30 min of storage and 28% after the 60 min mark. It should be noted though that the LC droplet configuration remained mostly radial during the storage experiment.

On the other hand, SDS-coated droplets did not perform well during storage. For R = 20, as depicted in Figure 4, during the 30 min period, the droplets started coalescing, deforming the LC organization (PDI 69%). Moreover, after the one-hour period, larger and disoriented LC aggregations developed (PDI 64%). For R = 95, the opposite effect was observed; as time progressed during storage, the droplets experienced Ostwald ripening and droplets with smaller diameters emerged—PDI 52% at 30 min and 61% at 60 min.

A stable surface is important in order to sustain droplet integrity over time. Our experiments show that [C_12_MIM][Cl] prevents droplet shrinking and coalescence, when compared to SDS. The structure of the [C_12_MIM][Cl] headgroup and the Cl^−^ anions at the droplet surface probably contribute towards a stronger interface between the LC oil phase and the IL aqueous phase outside the droplet. Due to the delocalized electrons on the imidazole ring and the hydrogen bonding between imidazole rings [33], [C_12_MIM][Cl] offers richer possibilities of interaction at the surface of the droplet, when compared to the SDS headgroup. In addition, the Cl^−^ anions tend to adsorb at the droplet surface through electrostatic interactions with the cationic headgroups and further establish hydrogen bonds with the surrounding water, which could further promote droplet surface stabilization [34,35].

## 3. Materials and Methods

### 3.1. Materials

The nematic liquid crystal 5CB (4-Cyano-4’-pentylbiphenyl) was acquired from TCI chemicals (Shanghai, China). Ionic liquids from the imidazolium chloride family ([C_2_MIM][Cl], [C_4_MIM][Cl] (>98%), [C_6_MIM][Cl] (>98%), [C_8_MIM][Cl] (>98%), [C_10_MIM][Cl] (>98%), [C_12_MIM][Cl] (>98%)) and [C_4_MIM][DCA] (>98%) were acquired from Iolitec (Heilbronn, Germany). Sodium dodecyl sulfate (SDS) micropellets were acquired from NZYTech (Lisbon, Portugal).

### 3.2. Microfluidics Set-Up

All experiments were carried out at room temperature. The liquids were supplied to a three-inlet flow-focusing quartz microfluidic (Dolomite Microfluidics (3200130), large droplet junction chip; see Figure 1 for junction dimensions) with hydrophilic-coated inner channels, through three independent microfluidic flow sensors (MFS3, Elveflow, Paris, France) for precisely measuring their flow rates. The flow of the liquids to the chip was controlled by a microfluidic flow controller (OB1 MK3, Elveflow, Paris, France). The pressure levels were controlled by Elveflow software, which translated pressure levels into flow rates, measured by the flow sensors. PTFE and FEP tubing were used for the connections in the setup.

Formation of on-chip droplets was imaged using an Axiocam 503 color camera mounted on a Zeiss Axioskop 40. Droplet observation was performed in a Polarizing Optical Microscope (POM) using a Zeiss Axio Observer.Z1 microscope equipped with an Axiocam 503 color camera. Photos taken were processed by the ZEN 203 software. For all experiments, the middle inlet of the chip was used for the disperse phase (5CB), the two outer inlets were used for the continuous phase (ionic liquid aqueous solution) (Figure 1A). Three different ionic liquid concentrations in aqueous solution were tested 0.02 M, 0.2 M and 0.4 M. In the case of ILs [C_2_MIM][Cl], [C_4_MIM][Cl] and [C_6_MIM][Cl], the concentrations 0.5 M, 2.6 M, 7.5 M and 16 M were also tested, however, no stable droplets were observed for any of them.

An important factor to be considered in microfluidics experiments is the relative flow rates of the continuous and dispersed phases. They are critical in controlling the droplet size, even determining whether droplet formation can occur. Typically, as the ratio (R) of the continuous phase flow rate to the disperse phase flow rate increases, the droplet diameter decreases. Owing to R increasing, the continuous phase interrupts the stream of the disperse phase more often, resulting in smaller droplets. In this study, seven different ratios (R) were tested: 5, 20, 35, 50, 65, 80 and 95. As an example, R = 35 means that the flow rate of the continuous phase is approximately 35 times higher than the flow rate of the disperse phase. To generate the different ratios, we kept the disperse phase flow rate at 1 µL/min and varied the continuous phase flow rate between 5 and 95 µL/min.

### 3.3. Droplet Analysis

While running the microfluidics experiments, individual drops (containing droplets) formed at the end of the outlet tube were periodically collected onto a glass slide. For all experiments, two independent drops were collected per condition. A condition is defined as a set of parameters that include the IL and concentration used for the continuous phase, the LC used for the disperse phase and the ratio, R, of their corresponding flow rates. As an example, a condition can be defined as using [C_12_MIM][Cl] at a 0.02 M concentration as the continuous phase, 5CB as the disperse phase and 20 as the R. Per drop, three regions of interest (ROI) were analyzed using the POM. This only applies to conditions where droplets were formed. All the images were obtained using the same magnification (50×) and each ROI had an area of 2.3 mm^2^. Since six ROI in total were analyzed per tested condition, the overall sample area for each condition was determined as 13.8 mm^2^, allowing for the determination of droplet yield defined as number of droplets per mm^2^. Additionally, the polydispersity index (PDI) [14,15] was used to analyze the variability of droplet diameters. The PDI is defined as:

(1) PDI% = Standard deviation/Average droplet diameter × 100

Droplet stability upon storage is also an important factor when determining the best condition for droplet production. This was evaluated by collecting the droplets in a 1.5 mL test tube immediately after production and extracting them for POM analysis. Droplets were extracted at three different periods: 0 min (immediately after droplet collection), 30 min and 60 min. A pipette was used to extract 5 µL of droplets each time. POM photos were taken with crossed polarizers and in bright field.

Establishing this analysis protocol aided us in determining the condition that provided the best combination for droplet output, LC configuration, droplet size dispersity and droplet stability upon storage.

## 4. Conclusions

Liquid crystals (LCs) are extremely interesting dynamic systems for the design of fast and low energy-driven transductors for optical sensing purposes. One of the most convenient and reproducible ways to generate LC-based sensors, is to encapsulate them in droplets, with defined yet reversible director configurations, by microfluidics. Microfluidics allows the automated and high-throughput production of droplets using low fabrication and operation costs [36]. So far, surfactants as SDS have been the mostly reported molecules to generate stable LC droplets for sensing. However, there are other surfactant active molecules, such as ionic liquids with long alkyl chains, which can provide a much wider and richer chemical diversity in LC systems. Past works showed that imidazolium- based ILs could generate stable LC droplets by emulsion, although with uncontrolled size distribution [15,16]. This work aimed at exploring the potential of ILs to generate uniform and stable LC droplets with defined director configurations, and at high yield, using microfluidics.

Aqueous solutions of ILs from the imidazolium family with varying length in the alkyl chain were used as the continuous phase, as well as SDS for comparison purposes.

In this setup, ILs with short alkyl chains exhibited weak surfactant properties and did not facilitate droplet production. With increasing the alkyl chain length of the ILs, droplet formation occurred, but also the LC director configuration gradually improved towards radial profiles. [C_12_MIM][Cl] provided the best results for all the tested surfactants with regards to droplet monodispersity, yield and stability but also in achieving the preferred radial LC organization. Interestingly, [C_12_MIM][Cl] originated very uniform and stable LC droplets, both in terms of size and of radial director configuration, when compared to SDS which is considered the state-of-the-art surfactant for LC droplet formation. This may be attributed to the effect of the imidazolium ring which confers multiple opportunities to pack LC molecules inside the droplets, offering a much richer chemical environment than the polar head of SDS.

As such, this work unfolds new possibilities for the use of the imidazolium-based ILs, namely [C_12_MIM][Cl], as alternative molecules for the production of stable and oriented oil-in-water droplets produced by microfluidics.

## Figures and Tables

**Table 1:** Droplet diameters and director configurations observed for the liquid crystal droplets formed by the tested ionic liquids on various concentrations and flow ratios.

Ionic Liquid	Concentration (M)	R	Average Diameter (µm)	Std. Dev. (µm)	Comments	PDI (%)	Yield (Droplets/mm^2^)
**SDS**	0.02	5	107	19	Dominant radial configuration until R = 65, after which escaped radial is dominant	17	60
		20	110	35		12	27
		35	100	22		7	27
		50	86	30		14	19
		65	43	34		22	8
		80	56	33		16	11
		95	48	25		24	0
	0.2	5	103	12	Mixture of radial and escaped radial	33	28
		20	94	8		21	29
		35	91	13		19	19
		50	82	6		8	11
		65	81	6		8	8
		80	80	4		8	7
		95	79	8		8	6
**C_12_MIMCl**	0.02	5	102	10	Radial configuration across all ratios	10	40
		20	105	26		24	31
		35	94	16		17	35
		50	87	9		11	30
		65	85	8		10	21
		80	80	6		7	19
		95	74	6		8	21
	0.2	5	98	18	Mixture of radial and escaped radial	18	70
		20	93	22		23	25
		35	81	12		15	16
		50	75	11		15	15
		65	73	11		16	11
		80	72	12		17	9
		95	71	14		20	10
	0.4	5	115	43	Escaped radial	38	101
		20	120	57		47	30
		35	88	15		17	18
		50	79	12		15	13
		65	81	12		14	20
		80	78	23		29	6
		95	77	17		22	7
**C_10_MIMCl**	0.02	No droplets were formed					
	0.2	No droplets were formed					
	0.4	5	99	54	Mixture of different configurations	55	4
		20	115	56		49	6
		35	130	44		34	2
		50	131	37		28	1
		65	85	22		26	6
		80	101	26		26	3
		95	106	15		15	1
**C_8_MIMCl**	0.02	No droplets were formed					
	0.2	–	–	–	Mixture of different configurations	–	–
		20	190	58		31	2
		35	132	49		37	12
		50	126	52		41	26
		65	114	45		40	28
		80	98	41		42	15
		95	90	21		23	2
	0.4	–	–	–	Mixture of different configurations	–	–
		20	160	80		50	0
		35	132	87		66	3
		50	111	35		31	13
		65	96	30		31	13
		80	102	25		25	3
		95	103	31		30	21

## Data Availability

The data presented in this study is contained within this article and is supported by data in the Supplementary Materials.

## Supplementary Materials

The following are available online, Figure S1: POM images when using short alkyl chain ionic liquids for the continuous phase, Figure S2: Size comparison for droplets formed with [C_8_MIM][Cl], Figure S3: POM and BF images of droplets formed using [C_8_MIM][Cl], Figure S4: Size comparison for droplets formed with [C_10_MIM][Cl], Figure S5: POM and BF images of droplets formed using [C_10_MIM][Cl] at concentration 0.4 M, Figure S6: Size comparison for droplets formed with SDS, Figure S7: POM and BF images of droplets formed using SDS.
