# Supplementary material for: Stable and Oriented Liquid Crystal Droplets Stabilized by Imidazolium Ionic Liquids
Source: Molecules. 2021 Oct 5;26(19):6044. doi: 10.3390/molecules26196044 (PMC8512111; doi:10.3390/molecules26196044)
Supplement: Supplementary file 1 [file molecules-26-06044-s001.zip › molecules-1386956-supplementary.pdf]

Electronic Supplementary Information

Stable and Oriented Liquid Crystal Droplets Stabilized by Imidazolium Ionic Liquids

Efthymia Ramou[1,2Ψ], Guilherme Rebordão[1,2Ψ], Susana I. C. J. Palma[1,2Ψ] and Ana C. A. Roque[1,2*]

[1] Associate Laboratory i4HB- Institute for Health and Bioeconomy, School of Science and Technology, NOVA University Lisbon, 2819-516 Caparica, Portugal
[2] UCIBIO – Applied Molecular Biosciences Unit, Department of Chemistry, School of Science and Technology, NOVA University Lisbon, 2819-516 Caparica, Portugal
* Correspondence: cecilia.roque@fct.unl.pt
Ψ Authors contributed equally

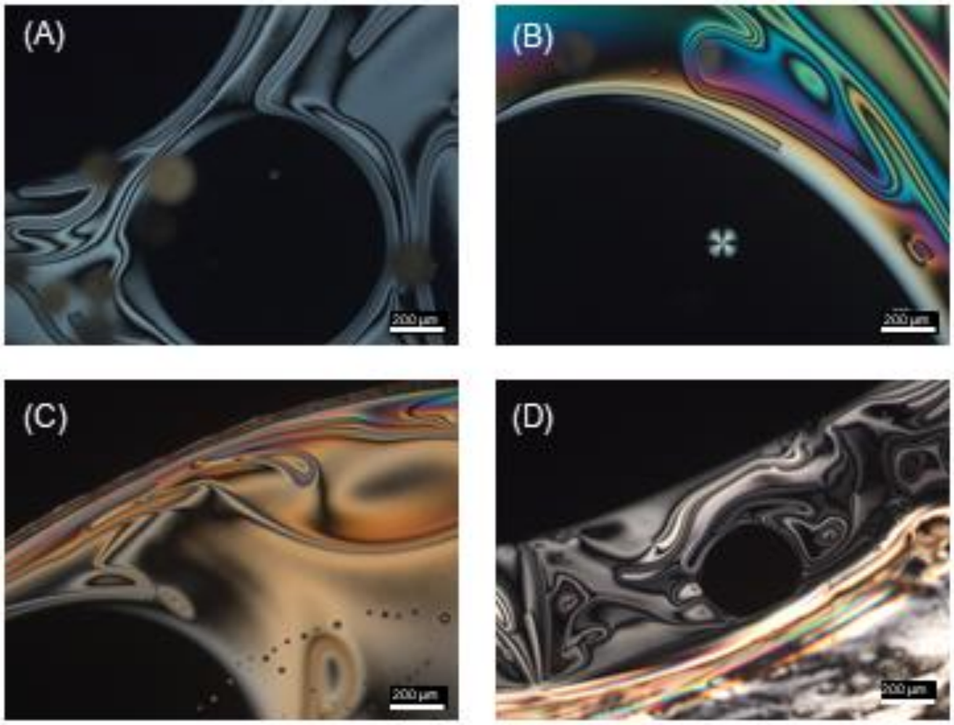

Figure S1. POM images showing separation of phases when using (A) [C_2MIM][Cl], (B) [C_4MIM][DCA], (C) [C_4MIM][Cl], (D) [C_6MIM][Cl] for the disperse phase in the microfluidics experiments.

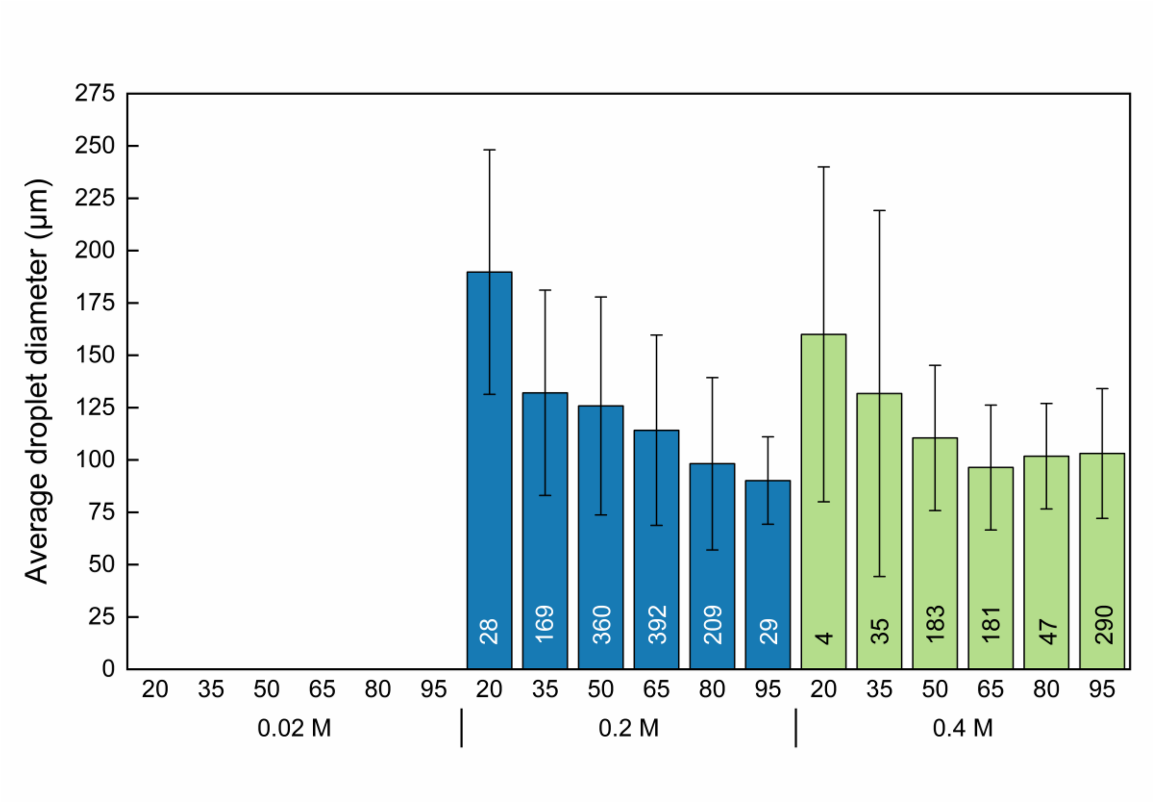

Figure S2. Size comparison between droplets formed with [C_8MIM][Cl] at concentrations 0.02 M, 0.2 M, and 0.4 M, respectively. On the x axis the different tested flow ratios are represented. The number of analyzed droplets is shown inside the respective bars. No droplets were produced when using [C_8MIM][Cl] at a 0.02 M concentration.

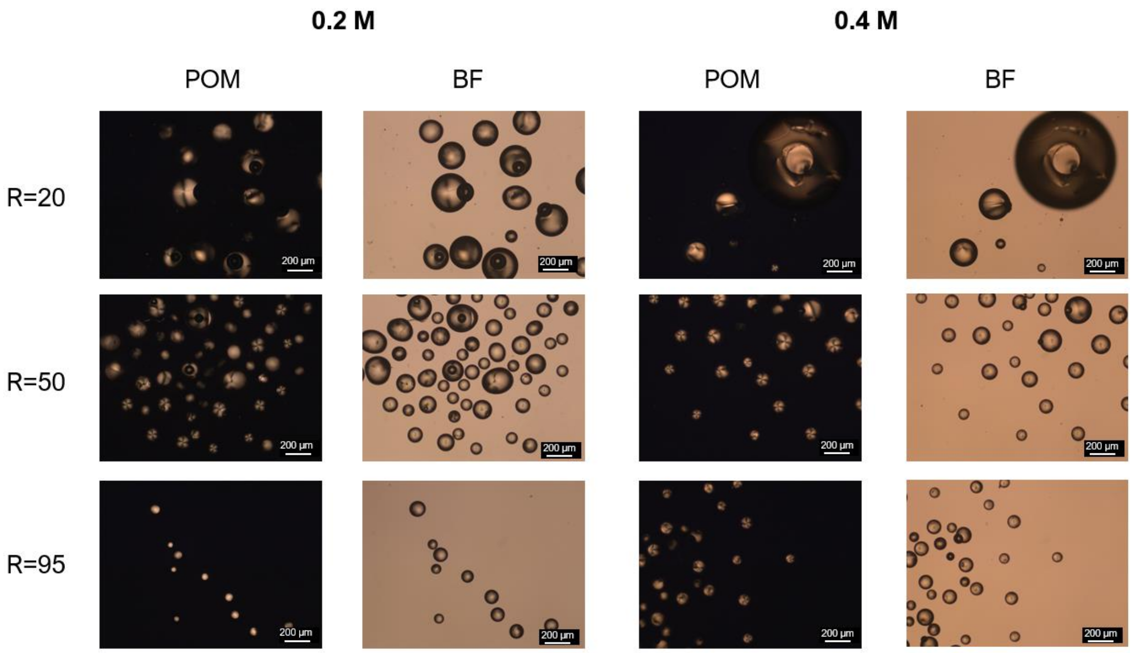

Figure S3. POM and BF images of droplets formed using [C_8MIM][Cl] at concentrations 0.2 M and 0.4 M, respectively. For clarity reasons, only 3 different Rs are displayed here: 20, 50, and 95.

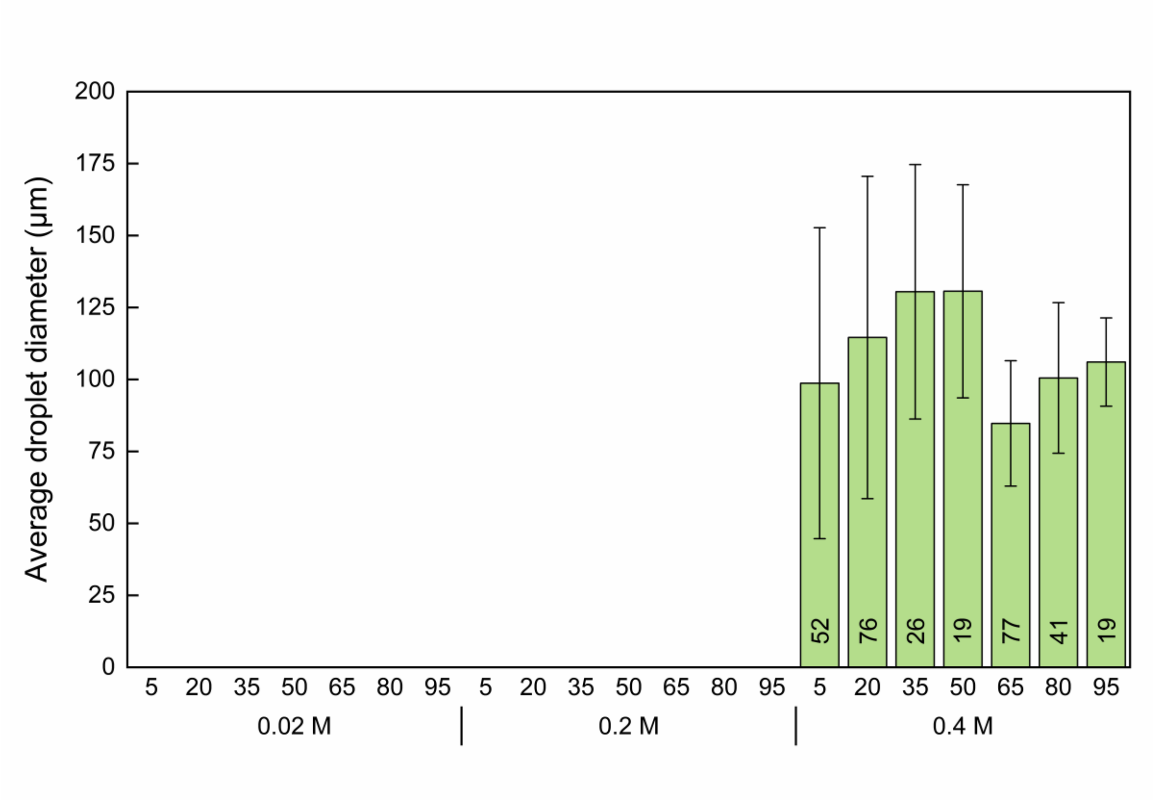

Figure S4. Comparison between droplets formed using [C$_{10}$MIM][Cl] at concentrations 0.02 M, 0.2 M, and 0.4 M, respectively. On the x axis the different tested flow ratios are represented. No droplets were produced when using concentrations 0.02 M and 0.2 M. The number of analyzed droplets is shown inside the respective bars.

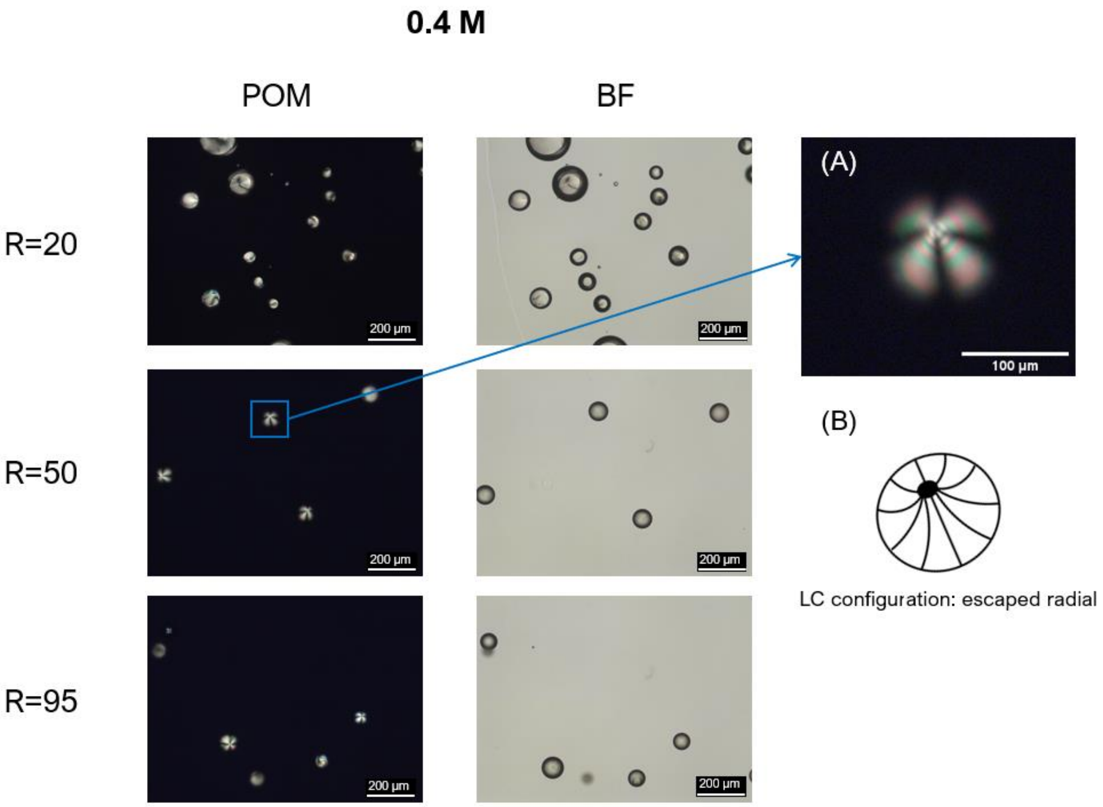

Figure S5. POM and BF images of droplets formed using $[C_{10}MIM][Cl]$ at concentration 0.4 M. For clarity reasons, only 3 different Rs are displayed: 20, 50, and 95. (A) Droplet close-up. (B) Illustration showing the escaped radial configuration of the droplet. [1]

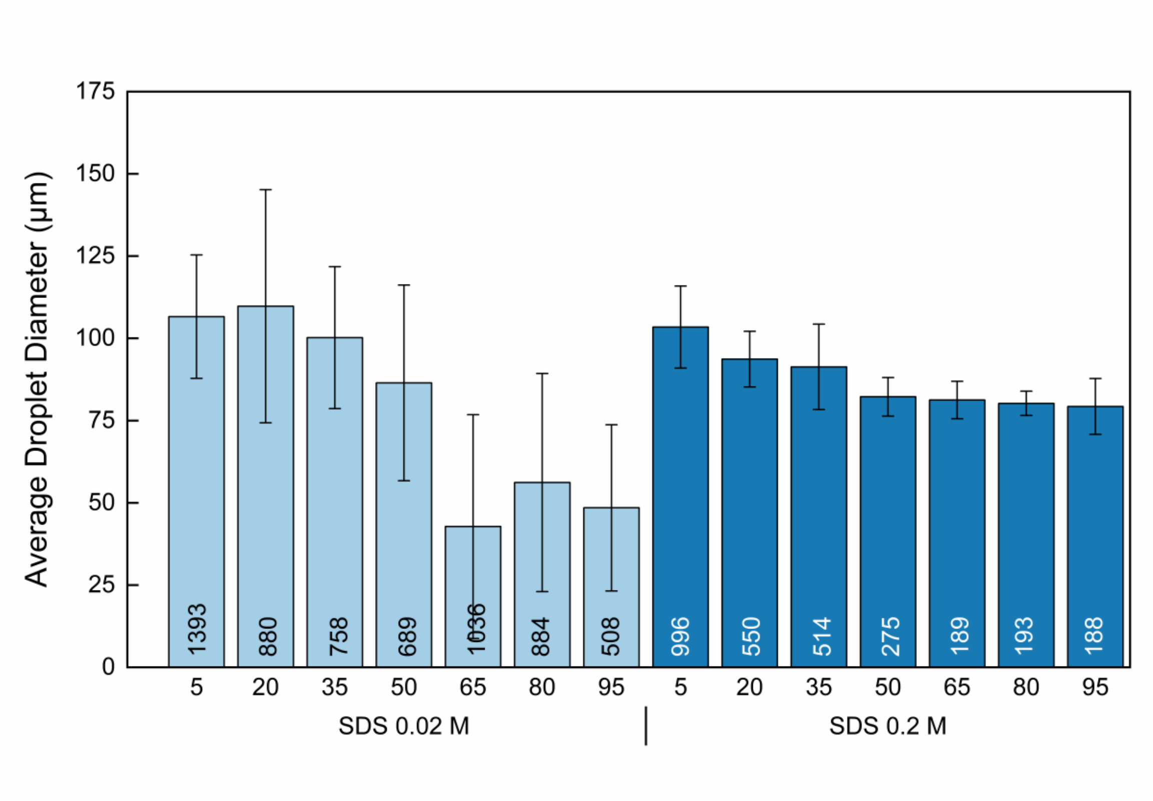

Figure S6. Size comparison between droplets formed using SDS at concentrations 0.02 M and 0.2 M, respectively. On the x axis the different tested flow ratios are represented. The number of analyzed droplets is shown inside the respective bars.

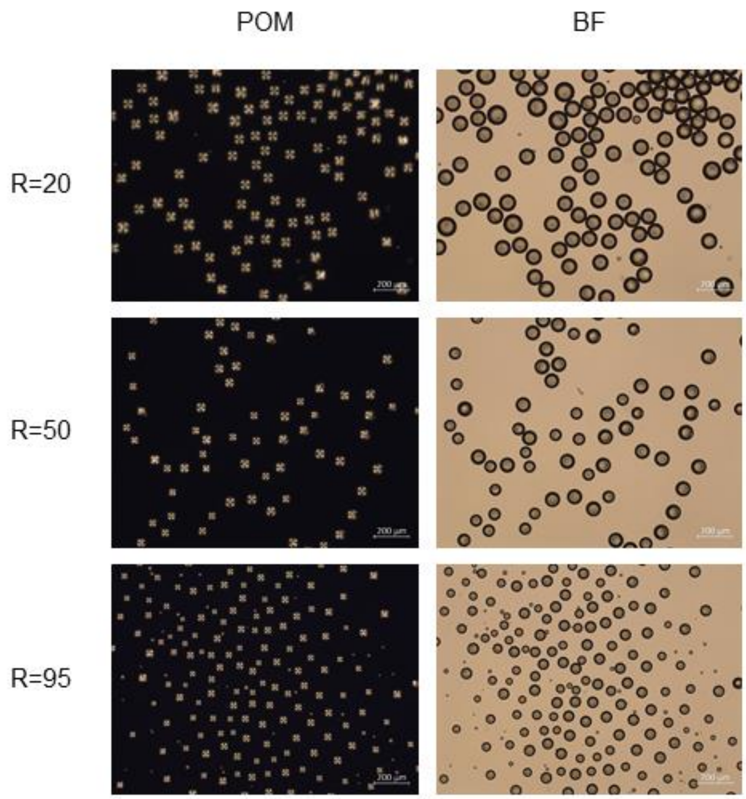

Figure S7. POM and BF images of droplets formed using SDS at concentration 0.02 M. For clarity reasons, only 3 different Rs are displayed: 20, 50, and 95.

References

1. Gupta, J.K.; Zimmerman, J.S.; De Pablo, J.J.; Caruso, F.; Abbott, N.L. Characterization of adsorbate-induced ordering transitions of liquid crystals within monodisperse droplets. *Langmuir* **2009**, *25*, 9016–9024, doi:10.1021/la900786b.
